# Dietary and environmental modulation for the gut environment: yogurt promotes microbial diversity while chloride hot springs improve defecation status in healthy adults

**DOI:** 10.3389/fnut.2025.1609102

**Published:** 2025-06-30

**Authors:** Jungmi Choi, Midori Takeda, Shunsuke Managi

**Affiliations:** Urban Institute, Kyushu University, Fukuoka, Japan

**Keywords:** hot spring, yogurt, gut microbiota, short-chain fatty acid, lifestyle intervention

## Abstract

The gut microbiome plays a central role in human health and can be shaped by both dietary and environmental factors. While yogurt has been widely studied for its ability to modulate the gut microbiota as a dietary factor, the effects of chloride hot spring bathing as an environmental factor remain largely unexplored. This randomized, controlled trial investigated the individual and combined effects of yogurt consumption and chloride hot spring bathing on gut microbiota, fecal metabolites, and defecation function in healthy adults. 47 participants (39 eligible participants + 8 additional recruits) were randomly assigned to one of three groups: control, yogurt only, or yogurt plus hot spring bathing. Over a four-week period, participants in the yogurt groups consumed 180 g of yogurt daily, and those in the hot spring group additionally bathed in a chloride-rich hot spring at least every 2 days. Fecal samples and defecation status questionnaires were collected before and after the intervention. Gut microbiota profiles were analyzed using 16S rRNA gene sequencing, and short-chain fatty acids (SCFAs) were measured by gas chromatography–mass spectrometry (GC–MS). Yogurt consumption significantly increased gut microbial diversity, as shown by higher Shannon index, observed ASVs, and Faith’s phylogenetic diversity, with notable enrichment of beneficial taxa such as *Akkermansia*. A significant reduction in formic acid levels was also observed in the yogurt group, while overall SCFA profiles remained unchanged. Although no significant microbiota or metabolite shifts were detected in the yogurt + hot spring group, it showed the greatest numerical improvement in defecation scores. These findings suggest that accessible lifestyle interventions, such as dietary modification and hot spring bathing, can positively influence gut health and may serve as practical strategies for promoting overall well-being.

## 1 Introduction

The gut microbiome plays a fundamental role in maintaining human health (1), influencing various physiological processes such as digestion (2), metabolism (3), immune regulation (4, 5), and even neurological functions (6). Recent research has highlighted the dynamic nature of the gut microbiota, emphasizing how diet, environmental factors, and lifestyle choices collectively shape its composition and function (7, 8). Among dietary factors, fermented foods have been extensively studied for their ability to modulate the gut microbiota (9). Yogurt, in particular, is consumed worldwide as a source of probiotics and primarily contains beneficial bacterial strains like *Lactobacillus* and *Bifidobacterium* (10). Numerous studies demonstrate that regular consumption of yogurt can promote the growth of beneficial gut bacteria (11), enhance microbial diversity (12), improve gut barrier integrity (13), and contribute to overall gastrointestinal health (14). Additionally, probiotics in yogurt have been shown to influence systemic immune responses (15), reduce inflammation (16), and even play a role in mental health through the gut-brain axis (17).

Despite the well-established benefits of yogurt consumption on modulating the gut microbiota, the influence of environmental factors, particularly thermal bathing, on the gastrointestinal microbial ecosystem remains relatively underexplored.

Hot spring bathing has been practiced for centuries as a traditional approach to health promotion, and modern research increasingly supports its potential therapeutic effects. Evidence from clinical and epidemiological studies indicates that immersion in mineral-rich hot spring water may help regulate not only for relaxation and mental health (18) but also musculoskeletal pain (19), skin disorders (20, 21), and metabolic disease (22–24). While the physiological benefits of hot spring bathing have been well documented, emerging evidence suggests that thermal bathing may also influence the gut microbial ecosystem. Recent findings indicate that repeated exposure to certain types of mineral-rich hot springs may modulate the composition of the gut microbiota. For instance, a previous study reported a significant increase in the relative abundance of *Bifidobacterium bifidum* following seven consecutive days of bathing in a bicarbonate spring (25). These observations imply that the impact of hot springs may extend beyond surface-level physiological effects, potentially exerting systemic influence through pathways involving the gut microbiome.

In Japan, hot springs are classified based on their mineral content, and among them, chloride springs are one of the widely available and utilized types (26, 27). Chloride springs, as defined by the Japanese Ministry of the Environment (26, 27), contain ≥1 g/kg of chloride ions (Cl^−^) and are characterized by their salt-rich composition. This high salinity springs, due to their mineral content, have traditionally been associated with improved circulation and general well-being. However, while these hypotheses are intriguing, empirical evidence supporting a direct effect of hot spring bathing on gut microbiota remains limited. Furthermore, previous research on chloride springs suggests that, despite their physiological benefits, they do not induce significant alterations in gut microbiota composition when used in isolation (25). This finding raises important questions regarding the extent to which external environmental exposures, such as hot spring bathing, contribute to gut microbial modulation.

Given the established effects of yogurt consumption on gut health and the potential, albeit unclear, systemic effects of hot spring bathing, it is plausible that the combination of these two interventions could produce synergistic effects, enhancing the modulation of gut microbiota beyond what either intervention could achieve alone.

This study aims to assess the isolated and combined effects of yogurt consumption and hot spring bathing on gut microbiota. By systematically evaluating whether yogurt consumption can enhance the potential microbiota-related benefits of hot spring bathing, we seek to provide new insights into the interplay between dietary and environmental factors in shaping microbial diversity and function. This investigation will not only contribute to a deeper understanding of lifestyle-based approaches for improving gut health but may also offer novel perspectives on how traditional wellness practices can be integrated with modern nutritional strategies to optimize human well-being.

## 2 Materials and methods

### 2.1 Ethics approval and consent to participate

This study was approved by the Ethical Committees of Urban Institute (permission number: 230807–01). All participants provided written informed consent before enrollment in the study. The study was conducted in accordance with the Declaration of Helsinki.

### 2.2 Study subjects and bathing procedures

**Figure 1:**
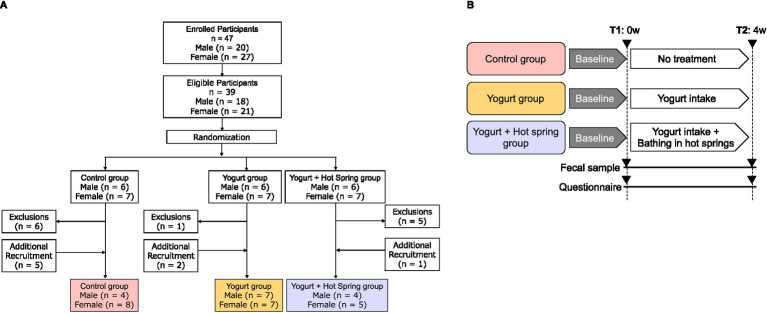
Flowchart of the study population. **(A)** Participants were screened based on inclusion and exclusion criteria. The final analysis included 39 individuals, and the numbers of participants are listed for each group. **(B)** Clinical trial design and time points of measurement. Participants were randomly assigned to one of three groups: Control, Yogurt, or Yogurt + Hot Spring. Each group followed their allocated intervention for 4 weeks. Measurements were taken at two time points: T1 (pre-intervention, baseline) and T2 (post-intervention, after 4 weeks of intervention) to evaluate changes in outcomes.

This study was a randomized, controlled trial conducted over 4 months, from September to December 2023. A total of 47 healthy adult participants (men and women) aged 20 to 65 years were recruited (Figure 1A). The inclusion criteria were as follows: (1) Who do not regularly consume yogurt. (2) Who have not bathed in a hot spring within the past 2 weeks. (3) Who are not currently taking antibiotics. (4) Who provided consent for the use of their personal data in research and for participation in the questionnaire survey. According to the inclusion criteria, 39 participants were randomly assigned to one of three intervention groups using stratified randomization to balance age and sex across groups: the control group, the yogurt group, and the yogurt + hot spring group. During the study period, 8 additional participants were recruited using the same inclusion criteria to replace those who dropped out due to scheduling or personal reasons. The same randomization procedure was applied for group assignment. Participants in the yogurt group consumed 180 g of Meiji Bulgaria Yogurt LB81 Low Sugar (Meiji, Tokyo, Japan), containing *Lactobacillus bulgaricus 2038 and Streptococcus thermophilus 1,131*, every evening after dinner. Detailed product information is available at: https://www.meijibulgariayogurt.com/en/product/LB81-low-sugar.html. Participants in the yogurt + hot spring group consumed the same 180 g of yogurt every evening after dinner and bathed in a chloride hot spring at least once every 2 days for at least 15 min. The hot spring used in this study was classified as a sodium chloride-type spring (NaCl), with a weakly acidic pH of 3.6. According to water analysis, it contained high concentrations of sodium ions (964.6 mg/kg) and chloride ions (1,446 mg/kg), as well as moderate levels of potassium (147.4 mg/kg), calcium (40.0 mg/kg), and sulfate (454.0 mg/kg). The water also contained metasilicic acid and boric acid. To minimize external influences, participants were instructed not to consume probiotic-containing foods and to avoid bathing in other hot springs during the study period. The intervention lasted for 4 weeks. Fecal samples and questionnaires were collected at two time points. T1 (before trial) was within 7 days prior to the start of the intervention, and T2 (after trial) was the first fecal sample collected after completing the intervention. Participants were instructed to maintain their usual diet and lifestyle throughout the study period (Figure 1B). The outcomes of the study included the occupancy of the gut microbiota, short-chain fatty acid concentration, and the defecation score, which reflects defecation status. After excluding samples with insufficient fecal volume, 35 participants were included in the final analysis. The detailed participant flow is illustrated in Figure 1A.

A questionnaire survey on defecation status of 14-item was developed to assess defecation status based on common symptoms reported in the literature and clinical practice. The questionnaire included items evaluating stool frequency, stool consistency, the sensation of incomplete evacuation, and the use of laxatives. The questions were designed based on literature review on functional constipation (28, 29). Participants rated their symptoms on a Likert scale from 1 (Yes), 2 (Sometimes), 3 (No). The full questionnaire is provided in Supplementary Table 1. Defecation score analysis was performed using data from 33 participants who fully responded to the questionnaire.

### 2.3 Fecal sample collection and processing

DNA was extracted from fecal samples using a previously described method (30). The V1-V2 variable region of the 16S rRNA gene was amplified using the universal bacterial primers 27F-mod (5’-AGRGTTTGATYMTGGCTCAG-3′) and 338R (5’-TGCTGCCTCCCGTAGGAGT-3′) with Tks Gflex DNA polymerase (Takara Bio Inc., Shiga, Japan) (31). The amplified DNA was sequenced in paired-end mode with 600 cycles using the MiSeq platform (Illumina, San Diego, CA, United States) according to the manufacturer’s protocol.

### 2.4 Measurement of SCFAs and organic acids

Fecal samples were lyophilized by a VD-800R lyophilizer (TAITEC) for more than 18 h. Lyophilized samples were homogenized with 3.0 mm zirconia beads at 1,500 rpm for 10 min using a ShakeMaster^®^ NEO homogenizer (Biomedical Sciences, Tokyo, Japan). A total of 10 mg of freeze-dried feces was used for analysis. For SCFAs and organic acids were measured using a 7,890 series gas chromatography-mass spectrometer (GC–MS, Agilent Technologies, CA, United States) following established protocols (30).

### 2.5 Bioinformatics analysis

16S rRNA gene analysis was performed using QIIME2 (version 2019.10). Primer sequences were removed using Cutadapt (option: -p-discard-untrimmed). Sequence data were processed with the DADA2 pipeline for denoising and quality filtering (option: -p-trunc-len-f 230 -p-trunc-len-r 130) (32). The resulting amplicon sequence variants (ASVs) were assigned to taxa using the Silva SSU Ref NR 99 database (version 132) with the “qiime feature-classifier classify-sklearn” command (33).

### 2.6 Statistical analysis

Statistical analyses were performed using R software (version 4.3.1) and Prism 9. The Wilcoxon matched-pairs signed-rank test was used to determine the change in the relative abundance of each gut microbiota and changes in the gut organic acids and SCFAs following before and after yogurt consumption and hot spring bathing. Group comparisons were conducted using the Kruskal-Wallis test, followed by Dunn’s *post hoc* test for pairwise comparisons. The false-discovery rate (FDR) method was used to correct *p* values for multiple testing, and *q* values were calculated using the Benjamini–Hochberg method. Statistical significance was set at *p* < 0.05 and *q* < 0.05.

## 3 Results

### 3.1 Participant characteristics

The numbers of 35 valid participants analyzed in this study are as follows: Control (*n* = 10), Yogurt (*n* = 14), and Yogurt + Hot Spring (*n* = 9) (Figure 1A). Baseline characteristics of the participants are presented in Table 1. The study population included 15 males and 20 females. The overall mean age was 39.6 years, with a mean age of 37.6 years for male and 42.1 years for female.

**Table 1:** Participant’s characteristics.

Variables	Categories	Control		Yogurt		Yogurt + hot spring	
		Male (*N* = 4)	Female (*N* = 8)	Male (*N* = 7)	Female (*N* = 7)	Male (*N* = 4)	Female (*N* = 5)
Age	Mean ± SD (min–max)	44.25 ± 5.19 (38–49)	38.13 ± 11.91 (25–63)	37.43 ± 11.39 (25–52)	45.14 ± 10.88 (40–58)	31.25 ± 17.17 (23–57)	44.20 ± 15.22 (26–61)

Data are presented as mean ± standard deviation for continuous variables.

### 3.2 Yogurt consumption enhances gut microbial diversity and alters taxonomic composition

**Figure 2:**
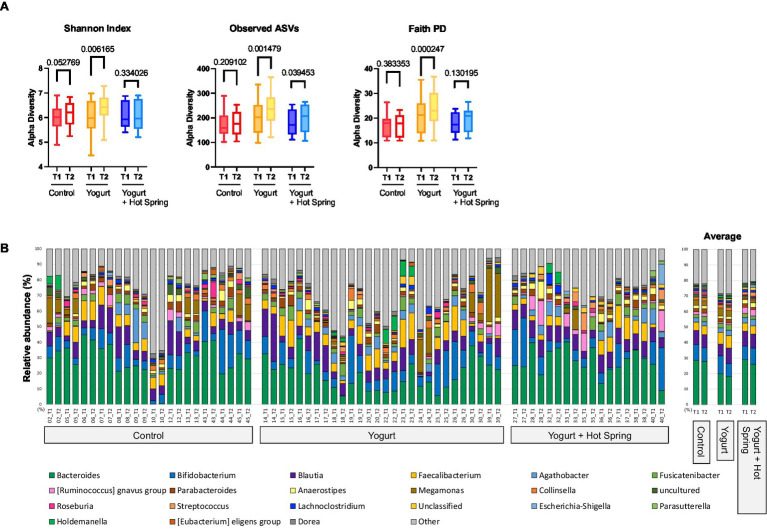
Effects of yogurt consumption and hot spring bathing on gut microbiota composition and diversity. **(A)** Alpha diversity of the gut microbiota before (T1) and after intervention (T2) estimated by Shannon, Observed ASVs and Faith’s PD (phylogenetic diversity) across three groups: Control, Yogurt, and Yogurt + Hot Spring. Diversity was assessed using alpha diversity indices, represented as boxplots. Statistical comparisons were performed to evaluate changes within and between groups. Exact false discovery rate (FDR)-corrected *q* values were presented in the figure. **(B)** Stacked bar plots showing the relative abundance of bacterial taxa at the genus level before and after intervention. Each color represents a different bacterial genus, and proportions indicate relative abundance. The three groups (Control, Yogurt, Yogurt + Hot Spring) are displayed, illustrating the compositional shifts in gut microbiota following intervention.

To investigate the effects of the interventions on gut microbial diversity and composition, 16S rRNA gene sequencing was performed, and taxonomic classification was conducted at the genus level (Supplementary Table 2). Alpha diversity was evaluated using the Shannon diversity index, observed amplicon sequence variants (ASVs), and Faith’s phylogenetic diversity (PD) index at baseline (T1) and post-intervention (T2) for each group (Figure 2A).

In the Yogurt group, significant increases were observed in Shannon diversity (*p* = 0.0031, *q* = 0.0062), observed ASVs (*p* = 0.0007, *q* = 0.0015), and Faith’s PD index (*p* = 0.0001, *q* = 0.0002), indicating enhanced microbial richness and phylogenetic diversity following yogurt consumption. No significant changes in alpha diversity were observed in the Control group. The Yogurt + Hot Spring group exhibited an increasing trend in all diversity indices; however, the differences were not statistically significant.

**Figure 3:**
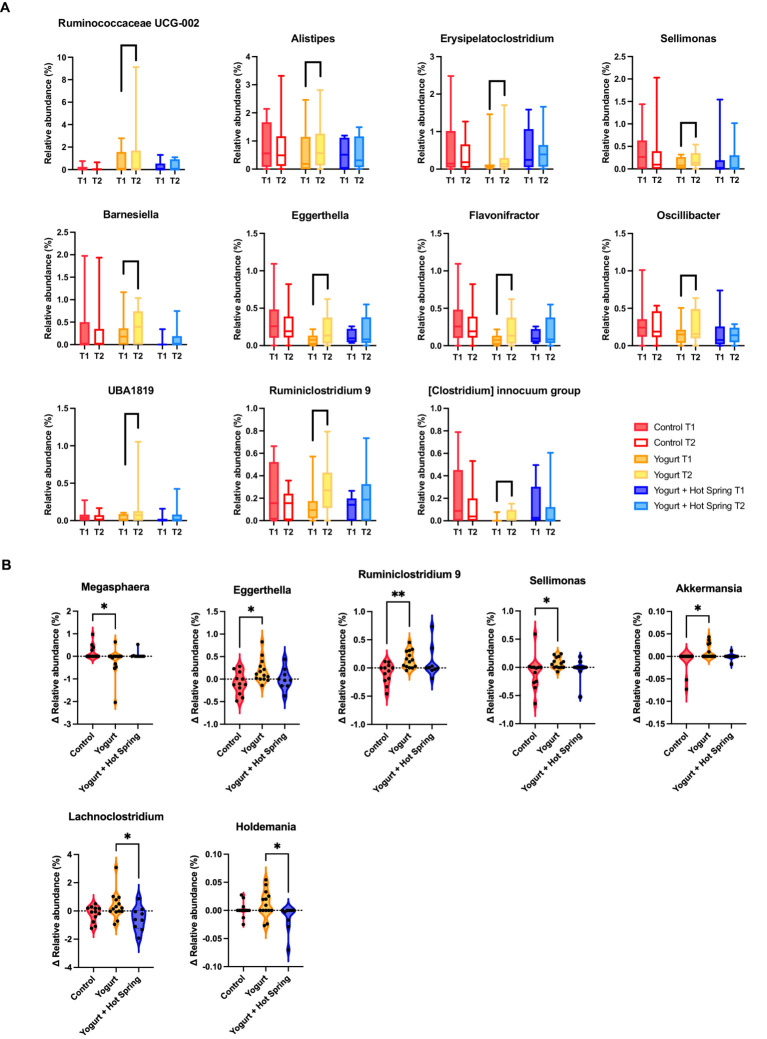
Changes in gut microbiota composition following yogurt consumption and hot spring bathing. **(A)** Boxplots representing gut bacterial genera that showed significant differences before and after the intervention within Yogurt group. Only bacterial genera with statistically significant changes (*p* < 0.05) are displayed. Statistical significance was determined using the Wilcoxon signed-rank test. **(B)** Difference in bacterial abundance (*Δ* abundance) across the three groups. Only bacterial genera that exhibited significant differences among the groups are shown. Statistical significance was assessed using the Kruskal-Wallis test, followed by Dunn’s *post hoc* test for pairwise comparisons. Statistical significance levels: *p* < 0.05 (*), *p* < 0.01 (**).

The top 20 genera in terms of relative abundance at baseline (T1) and post-intervention (T2) are shown in Figure 2B. The Control group remained relatively stable, although *Fusicatenibacter* exhibited a significant increase (Supplementary Table 3). Taxonomic shifts T1 and T2 in the Yogurt group are further illustrated in Figure 3A. Interestingly, the Yogurt group displayed significant increases in multiple genera, including *Ruminococcaceae UCG-002*, *Alistipes*, *Erysipelatoclostridium*, *Sellimonas*, *Barnesiella*, *Eggerthella*, *Flavonifractor*, *Oscillibacter*, *UBA1819*, *Ruminiclostridium 9*, and *[Clostridium] innocuum group* (*p* < 0.05, Wilcoxon signed-rank test). After false discovery rate (FDR) correction, several genera remained significant, including *Sellimonas*, *Eggerthella*, *Flavonifractor*, and *Ruminiclostridium 9* (Supplementary Table 3). In the Yogurt + Hot Spring group, no genera showed statistically significant changes in relative abundance between time points.

Inter-group comparisons (*Δ* Relative abundance) were conducted to assess differential responses to the interventions. The Kruskal-Wallis test, followed by Dunn’s *post hoc* analysis, revealed significant differences in the relative abundance of specific genera among the three groups (Figure 3B; Supplementary Table 4). Notably, *Megasphaera* was significantly reduced in the Yogurt group compared to the Control group (*p* < 0.05). In contrast, *Eggerthella*, *Ruminiclostridium 9*, *Sellimonas*, and *Akkermansia* were significantly more abundant in the Yogurt group than in the Control group (*p* < 0.05 or *p* < 0.01). Furthermore, *Lachnoclostridium* and *Holdemania* were significantly less abundant in the Yogurt + Hot Spring group compared to the Yogurt group (*p* < 0.05), suggesting a potential modulatory effect of combined interventions.

### 3.3 Effect of yogurt consumption and yogurt consumption with hot spring bathing on the gut metabolome and SCFAs

**Figure 4:**
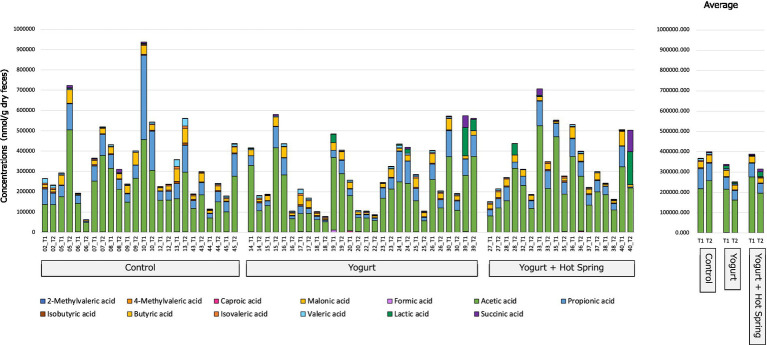
Effect on gut organic acids and SCFAs following yogurt consumption and hot spring bathing. This figure illustrates the changes in the gut organic acids and short-chain fatty acids (SCFAs) before and after the intervention in three groups: Control, Yogurt, and Yogurt + Hot Spring. Stacked bar plots representing the relative abundance of various gut metabolites, including SCFAs, before and after the intervention across different groups. Each color represents a distinct metabolite, and changes in composition are visualized over time within each group.

The gut metabolome, including short-chain fatty acids (SCFAs), was profiled T1 and T2 to evaluate the impact of yogurt consumption and its combination with hot spring bathing (Figure 4; Table 2).

**Table 2:** Statistical analysis of gut organic acids changes before and after intervention.

Organic acid	Control		Yogurt		Yogurt + hot spring	
	*p*-value	FDR *q*-value	*p*-value	FDR *q*-value	*p*-value	FDR *q*-value
Total SCFA	0.266	0.269	0.119	0.180	0.098	0.180
2-Methylvaleric acid	0.784	0.813	0.069	0.379	0.236	0.744
4-Methylvaleric acid	0.126	0.636	0.451	0.587	0.554	0.880
Caproic acid	0.784	0.813	1.000	1.000	0.286	0.744
Malonic acid	0.784	0.813	0.117	0.379	0.636	0.880
Formic acid	0.255	0.664	**0.028**	0.364	0.286	0.744
Acetic acid	0.170	0.636	0.117	0.379	0.155	0.744
Propionic acid	0.327	0.682	0.149	0.387	0.058	0.744
Isobutyric acid	0.367	0.682	0.851	0.922	0.722	0.880
Butyric acid	0.065	0.636	0.233	0.433	1.000	1.000
Isovaleric acid	0.196	0.636	0.851	0.922	0.813	0.880
Valeric acid	0.666	0.813	0.451	0.587	0.722	0.880
Lactic acid	0.813	0.813	0.208	0.433	0.787	0.880
Succinic acid	0.666	0.813	0.451	0.587	0.722	0.880

Organic acids with significant *p* values are shown in bold.

The Control group exhibited no notable changes in the relative abundance of SCFAs or other gut metabolites between T1 and T2. In the Yogurt group, a significant reduction in formic acid levels was observed after the intervention (*p* = 0.028); however, this difference did not remain significant following false discovery rate (FDR) correction (*q* = 0.364). No other SCFAs exhibited statistically significant changes in this group.

Similarly, in the Yogurt + Hot Spring group, no significant alterations in SCFA composition were detected between T1 and T2. Inter-group comparisons also did not identify any statistically significant differences in metabolite profiles among the three intervention groups (data not shown).

These findings suggest that yogurt consumption may exert modest effects on the gut metabolome, particularly influencing formic acid levels, whereas hot spring bathing alone or in combination with yogurt does not appear to significantly modulate SCFA composition.

### 3.4 Yogurt consumption and hot spring bathing tend to improve defecation status

Defecation status was assessed using a self-reported questionnaire, and a total defecation score was calculated for each participant by summing the responses to individual items. The average score across all participants was 35.68 ± 4.47, with lower scores indicating more severe defecation dysfunction.

**Figure 5:**
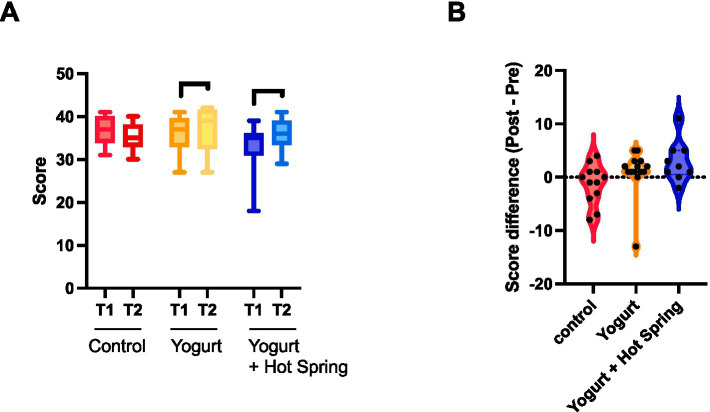
Effects of yogurt consumption and hot spring bathing on defecation scores. **(A)** Difference s in defecation scores within each group (Control, Yogurt, Yogurt + Hot Spring) before and after the intervention. Statistical significance was evaluated using the Wilcoxon signed-rank test to determine whether within-group differences were significant. **(B)** Difference in defecation scores (Δ score) across the three groups. Statistical analysis was conducted using the Kruskal-Wallis test followed by Dunn’s post hoc test for pairwise comparisons. Statistical significance levels: *p* < 0.05 (*).

The impact of the interventions on defecation scores was evaluated by comparing T1 and T2 values within each group (Figure 5A). An increase in total defecation scores was observed in both the Yogurt group and the Yogurt + Hot Spring group following the intervention, whereas no significant change was noted in the Control group.

Group differences in the change from baseline (*Δ* score) were further analyzed (Figure 5B). Although the Kruskal-Wallis test followed by Dunn’s *post hoc* test did not reveal statistically significant differences between groups, the Yogurt group demonstrated a trend toward greater improvement compared to the Control group. Moreover, the Yogurt + Hot Spring group (Mean ± SD, 2.889 ± 3.790) showed a numerically larger increase than both the Control (−1.250 ± 3.671) and Yogurt groups (1.000 ± 4.297).

Taken together, these findings suggest that yogurt consumption may help improve defecation status, and that hot spring bathing may have a potential additive effect. However, these trends did not reach statistical significance and require further investigation in larger cohorts.

## 4 Discussion

Novel insights into the distinct and combined effects of yogurt consumption and hot spring bathing on gut health were gained by evaluating changes in gut microbiota composition, fecal metabolomic profiles, and defecation function. The findings indicate that these interventions may influence the gut environment through complementary yet largely independent mechanisms.

First, yogurt consumption significantly increased gut microbial diversity, as indicated by elevations in Shannon diversity, observed ASVs, and Faith’s phylogenetic diversity (Figure 2A). Higher microbial diversity is often associated with enhanced gut ecosystem resilience and has been linked to improved metabolic and immune function (34). These findings underscore the potential of yogurt consumption to contribute to a more balanced and functionally robust gut microbiome.

Second, notable shifts in gut microbial composition were observed in the Yogurt group, including a significant increase in *Akkermansia* (Figure 3A). *Akkermansia* is a well-established commensal bacterium associated with gut barrier integrity (35), glucose homeostasis (36), and anti-inflammatory effects (37). Previous studies have reported that higher abundance of *Akkermansia*, correlates with improved metabolic outcomes in both clinical and preclinical models of obesity and metabolic syndrome (38–41). Due to its consistent association with metabolic health, *Akkermansia* has garnered considerable interest as a promising next-generation probiotic candidate. Our findings align with these reports, suggesting that yogurt consumption may promote gut health by increasing *Akkermansia* levels. In addition to *Akkermansia*, we observed significant increases in multiple genera such as *Ruminococcaceae UCG-002*, *Alistipes*, *Erysipelatoclostridium*, *Sellimonas*, *Barnesiella*, *Eggerthella*, *Flavonifractor*, *Oscillibacter*, and *Ruminiclostridium 9*. *Ruminococcaceae UCG-002, Alistipes, Flavonifractor*, *Oscillibacter*, are known SCFA producers (42–44) or *Sellimonas, Barnesiella*, *Eggerthella,* have been associated with gut immune modulation (45, 46), suggesting that yogurt may enhance gut function through indirect microbial reshaping. Furthermore, recent evidence suggests that modulation of the gut microbiota through dietary interventions such as yogurt may have implications beyond gastrointestinal health. For instance, Bhadani et al. discussed the gut microbiome’s potential role in orthopedic conditions and suggested that probiotics could serve as preventive or adjunctive strategies for managing inflammation-related disorders (47). These insights underscore the broader systemic relevance of gut microbial modulation by yogurt. Interestingly, despite yogurt’s known probiotic content, the relative abundance of *Bifidobacterium* and *Lactobacillus* did not significantly increase (10). This result suggests that yogurt consumption does not always lead to direct colonization of these bacteria and that its beneficial effects may instead arise from indirect modulation of existing microbial communities.

Third, in terms of gut metabolome, yogurt consumption was associated with a significant reduction in formic acid, while other SCFA levels remained unchanged. Given that *Akkermansia* has been linked to SCFA metabolism and gut epithelial health (48, 49), its increase might contribute to shifts in organic acid metabolism. However, the lack of significant changes in SCFA levels suggests that the bacterial taxa responsible for SCFA production did not exhibit major alterations, maintaining metabolic homeostasis. Moreover, the yogurt used in this study was a commercially available standard product, not enriched with high-dose probiotics, which may account for the limited impact on SCFA profiles.

Fourth, the combined yogurt and chloride rich hot spring intervention did not lead to significant changes in gut microbiota composition or organic acid levels. Our findings indicate that chronic chloride hot spring exposure may have limited impact on gut microbial composition in healthy individuals. One potential explanation is that prolonged exposure leads to physiological adaptation, reducing microbial fluctuations that might be observed in short-term interventions. Moreover, previous research using the same chloride-type hot spring reported no significant changes in gut microbiota, suggesting that this particular hot spring composition may have limited direct effects on these outcomes. Additionally, hot spring bathing may influence defecation function through non-microbial mechanisms, such as modulation of the autonomic nervous system or stress reduction, rather than through direct changes in microbial composition. This is supported by our observation that despite not statistically significant microbial changes, the yogurt + hot spring group demonstrating the largest improvement in defecation scores. This suggests that both interventions may positively influence defecation motility potentially via distinct mechanisms, such as microbial modulation in the case of yogurt, and relaxation or autonomic regulation in the case of chloride hot spring bathing rather than through a synergistic effect.

A major strength of this study lies in its randomized intervention design, which enabled a controlled evaluation of the individual and combined effects of yogurt consumption and hot spring bathing. Additionally, the combination of gut microbiota profiling, organic acids analysis, and defecation status assessments provides a comprehensive perspective on gut health. Nevertheless, several limitations must be acknowledged. First, the relatively small sample size may have limited the statistical power to detect subtle microbial or organic acids changes, particularly in the Yogurt + Hot Spring group. Second, although the four-week intervention period was sufficient to observe defecation-related outcomes, it may have been too long to capture acute microbial fluctuations associated with hot spring bathing. Previous studies suggest that short-term thermal exposure can modulate immune responses (50); however, the physiological effects of long-term exposure remain poorly understood. It is possible that adaptation to repeated bathing reduced the detectability of dynamic changes in gut microbiota and organic acids. Third, although improvements in defecation function were observed, key mechanistic parameters such as stress levels, gut motility markers, and immunological profiles were not assessed, limiting our ability to elucidate the underlying biological pathways. Fourth, the yogurt used in this study was a conventional product, and probiotic-enriched yogurt formulations may produce different outcomes. Future studies should consider larger sample sizes, different durations or patterns of thermal exposure, extended yogurt intervention periods and different yogurt formulations, and the inclusion of functional markers to better understand the complex interactions between lifestyle factors and gut health.

Yogurt consumption significantly improved gut microbial diversity and increased the abundance of beneficial taxa such as *Akkermansia*, while also contributing to improved defecation scores. Hot spring bathing, although not associated with significant microbial or metabolic shifts, was similarly linked to enhanced defecation function. These findings highlight the potential of both dietary and environmental interventions to support gut health through complementary pathways, offering accessible strategies for promoting overall well-being.

## Data Availability

The datasets presented in this study are available in the FigShare repository, DOI: 10.6084/m9.figshare.28758761.v1 (51).
